# Effects of different weekly frequencies of combined training on blood pressure variability in aging adults with hypertension

**DOI:** 10.3389/fresc.2026.1925760

**Published:** 2026-10-19

**Authors:** Lucas Betti Domingues, Vinícius Mallmann Schneider, Leandro Carpes, Sandra C. Fuchs, Rodrigo Ferrari

**Affiliations:** 1 Postgraduate Program in Cardiology and Cardiovascular Science, School of Medicine, Universidade Federal do Rio Grande do Sul, Porto Alegre, RS, Brazil; 2 Sports and Exercise Training Study Group, Clinical Research Center, Hospital de Clínicas de Porto Alegre, Porto Alegre, RS, Brazil; 3 Faculty of Physical Education, Universidade do Extremo Sul Catarinense, Criciúma, SC, Brazil; 4 Postgraduate Program in Human Movement Sciences, School of Physical Education, Universidade Federal do Rio Grande do Sul, Porto Alegre, RS, Brazil

**Keywords:** ambulatory blood pressure monitoring (ABPM), blood pressure variability (BPV), combined training, dose-response, exercise training, hypertension, older adults

## Abstract

**Background:**

Elevated blood pressure (BP) variability is associated with cardiovascular mortality, independent of mean BP levels. While exercise may influence short-term BP variability, evidence remains inconclusive, and the impact of different weekly training frequencies is unknown.

**Purpose:**

To compare the effects of a pragmatic combined training (CT) intervention performed twice vs. four times per week on short-term BP variability in older adults with hypertension.

**Methods:**

In this randomized clinical trial, men and women aged 50–80 years were assigned to CT twice (CT-2) or four (CT-4) times per week for 12 weeks. The protocol adopted a pragmatic approach, utilizing a weekly exercise dosage: 120–160 min/week and intensity by perceived exertion (4–6). Total volume and overload were matched between groups. Short-term BP variability was assessed via average real variability (ARV) and standard deviation (SD) for 24-hour, daytime, and nighttime periods.

**Results:**

Forty-seven participants (CT-2: *n* = 27; CT-4: *n* = 20) with ≥80% attendance were analyzed. No significant group × time interactions were observed. However, both CT-4 and CT-2 significantly reduced 24-hour systolic ARV (CT-4: −2 ± 1 mmHg, *P* = 0.003; CT-2: −1 ± 1 mmHg, *P* = 0.032) and daytime systolic ARV (CT-4: −3 ± 1 mmHg, *P* = 0.008; CT-2: −2 ± 1 mmHg, *P* = 0.019). Only CT-2 showed reduced daytime diastolic ARV (−1 ± 1 mmHg; *P* = 0.016). SD-based indices remained unchanged.

**Conclusion:**

Twelve weeks of combined training was not associated with statistically significant differences in short-term BP variability between training frequency groups (2 vs. 4) among aging adults with hypertension. Reductions in short-term systolic BP variability were observed over time in both training groups.

**Clinical Trial Registration:** https://clinicaltrials.gov/study/NCT04218903, NCT04218903.

## Introduction

Blood pressure (BP) is a dynamic physiological variable whose primary function is to adjust in response to cardiovascular demands (1). In pathological states such as hypertension, these fluctuations may become exaggerated (i.e., increased BP variability), leading to BP variability being recognized as a relevant complementary marker in cardiovascular risk assessment, adding independent prognostic value beyond mean BP levels (2, 3). Short-term BP variability is assessed through ambulatory BP monitoring and reflects the fluctuations in BP that occur over 24 h, including daytime, nighttime, and during daily living activities (4). This component of BP variability is particularly important because it is associated with key physiological mechanisms involved in BP regulation, such as baroreflex sensitivity, arterial stiffness, and flow-mediated dilation, as well as with overall physical fitness levels (5, 6).

Current evidence suggests that combined training, incorporating both aerobic and resistance exercises, is a cornerstone intervention for simultaneously reducing blood pressure (BP) levels and improving health-related physical fitness (7, 8). Most exercise intervention studies in individuals with hypertension have primarily focused on reducing mean BP levels, while few have specifically targeted short-term BP variability (8). A previous randomized clinical trial compared the effects of aerobic vs. combined training, reporting that combined training reduced 24-hour and nighttime systolic BP variability, whereas aerobic training only reduced 24-hour systolic BP variability (9). Conversely, another trial involving 10 weeks of combined training (3 sessions/week) in hypertensive women found that while mean BP levels decreased, short-term BP variability remained unchanged (10). These contrasting findings highlight the current inconsistencies in the evidence regarding the effects of exercise on BP variability. Such discrepancies may be explained, at least in part, by variations in acute training variables such as intensity, volume and frequency, which can substantially modulate the cardiovascular impact of an exercise training program (11, 12). In particular, training frequency seems to be highly relevant in the management of hypertension since the cumulative effects of acute BP reductions induced by exercise (post-exercise hypotension) may contribute to long-term adaptations in BP (13). Nevertheless, no study has directly compared the effects of equal total weekly training volume distributed across different weekly frequencies on short-term BP variability.

Given the current gaps in the literature regarding the impact of combined training on short-term BP variability and the lack of studies exploring the influence of training characteristics (i.e., different weekly frequencies), the primary objective of this study was to compare the effects of a combined training intervention performed either twice or four times per week on short-term BP variability in older adults with hypertension.

## Materials and methods

### Experimental design

This study is a secondary analysis of the Resistance Plus Aerobic Training Using Different Weekly Frequencies and Hypertension Study (RADAH Study), a superiority, randomized parallel-group trial with an equal allocation ratio (1:1). Random sequence generation, allocation concealment, and implementation followed the trial protocol. The parent trial evaluated the differential effects of combined training performed twice or four times per week on 24-hour BP and physical fitness in older adults with hypertension (14), and its full protocol has been published previously (15). Here, we investigated the effect of training frequency (i.e., two vs. four times per week) on short-term BP variability. Preliminary findings from this study were previously presented and published as a conference abstract (16).

The trial protocol was approved by the Ethics Committee of Hospital de Clínicas de Porto Alegre (reference number: 2019-0572) and registered on ClinicalTrials.gov under the code NCT04218903 (https://clinicaltrials.gov/study/NCT04218903). This report was prepared in accordance with the Consolidated Standards of Reporting Trials (CONSORT) guidelines and their extension to non-pharmacological clinical trials (17, 18).

### Participants

Participants who achieved an attendance rate of 80% or greater for either four times or twice weekly combined training interventions were included in this analysis. Men and women were eligible if they had a previous diagnosis of hypertension, were 50–80 years old, were using up to three classes of antihypertensive medications, and had not participated in structured exercise programs during the previous three months. Participants were excluded if they had physical or musculoskeletal injuries that limited the performance of the proposed training protocols; a history of cardiovascular events (e.g., acute myocardial infarction, angina, or stroke) within the previous 24 months; heart failure classified as New York Heart Association class III or IV; chronic diseases such as cancer, dialysis-dependent kidney disease, multiple sclerosis, or Parkinson's disease; a body mass index ≥40 kg/m^2^; or diabetes mellitus with target organ damage (15).

All ethical principles, including confidentiality and data protection measures, as outlined in the Declaration of Helsinki and Resolution No. 466/12 of the Brazilian National Health Council, were followed to ensure the participants' privacy.

### Settings

The trial was conducted between August 2022 and December 2024 at the Clinical Research Center of the Hospital de Clínicas de Porto Alegre, and at the Exercise Research Laboratory of the Universidade Federal do Rio Grande do Sul, both located in southern Brazil.

### Combined training intervention

The combined training intervention took place on an outdoor athletic track, with sessions scheduled either in the morning or late afternoon, based on the participants’ preferences. In both the CT-2 and CT-4 groups, participants engaged in the same total training volume and overload each week, which included minutes of exercise, resistance exercises (number of sets, repetitions, intervals between sets and repetitions), aerobic exercise modality, and the relative intensity of resistance and aerobic training. The sole distinction between the groups was the frequency of training sessions per week, with participants in the CT-2 group attending two sessions per week and those in the CT-4 group attending four.

Participants in both groups performed combined training sessions that included resistance and aerobic components in sequence, beginning with body-weight-based resistance exercises immediately followed by aerobic exercise (walking or running). All sessions were conducted under the supervision of researchers and trained professionals. Participants in the CT-4 group engaged in four sessions per week, each comprising 10–15 min of resistance training (1–4 sets of 10–15 repetitions per exercise) followed by 20–25 min of aerobic exercise. Participants in the CT-2 group performed two sessions per week, each consisting of 20–30 min of resistance training (1–4 sets of 10–15 repetitions) followed by 40–50 min of aerobic exercise. The full training periodization protocol has been previously published (15).

The intensity of the training sessions was monitored using a rate of perceived exertion (RPE) scale (Borg's modified CR10 scale) (19) and remained consistent throughout the intervention. The target exercise intensity corresponded to approximately 50%–60% of estimated one-repetition maximum (1RM) for the resistance exercises, and 60% of estimated peak oxygen uptake (VO_2_peak) for aerobic exercises, aligning with RPE scores of 4–6 on the CR10 scale. Bodyweight resistance exercises included push-ups performed on street workout bars or on the floor, inverted rows using an overhead strap attached to a bar, calf raises on a step, squats, and abdominal exercises on floor mats. Exercise modifications were applied as necessary to maintain the prescribed RPE.

### Blood pressure assessment

Evaluations were conducted at baseline and endpoint (after 12 weeks of intervention) in CT-4 and CT-2 arms. Ambulatory blood pressure monitoring (ABPM) was performed using an automatic oscillometric device (ABP 2400, Mortara, Milwaukee, WI, USA). A valid ABPM recording was defined as having at least 70% successful readings, including a minimum of 20 daytime and 7 nighttime measurements. Among these recordings, those with at least 48 valid readings over 24 h were considered optimal and were used to estimate short-term BP variability (20). The default daytime period was set from 6:00 a.m. to 9:59 p.m., and the nighttime period from 10:00 p.m. to 5:59 a.m. Participants were instructed to complete a diary to record daily activities, any device issues, the timing of antihypertensive medication use, and their actual sleep and wake-up times. ABPM data were subsequently adjusted according to the self-reported sleep and wake-up times. Outcome assessors were blinded to group allocation.

BP variability was calculated using the ABPM data. All valid systolic and diastolic BP measurements were exported to a Microsoft Excel 2013 worksheet (Microsoft Corp., Redmond, WA, USA) for analysis. BP variability was calculated using two methods: standard deviation (SD) and average real variability (ARV). The SD of BP measurements was calculated for each participant and reported separately for the 24-hour, daytime, and nighttime periods. The ARV index, weighted for the time intervals between consecutive readings, was also calculated for systolic and diastolic BP across the same periods. The SD index reflects the dispersion of BP values around the mean and does not account for the temporal sequence of fluctuations. In contrast, the ARV represents the average absolute difference between successive BP readings, thus capturing reading-to-reading BP fluctuations.

### Statistical methods

Data were entered in duplicate by two independent researchers into a Microsoft Excel worksheet (Microsoft Corp., Redmond, WA, USA). In the case of any identified discrepancies, they were resolved by consulting the original research records. The assumption of normality was assessed using the Shapiro–Wilk test combined with visual inspection of histograms and Q-Q plots. Continuous variables were presented as mean ± standard deviation, while categorical variables were reported as absolute and relative frequencies. Short-term BP variability was expressed as mean ± standard error (SE) with 95% confidence intervals. Baseline comparisons of continuous variables were conducted using independent samples *t*-tests, while the chi-squared test was applied for categorical variables. Generalized estimating equation models were used to evaluate the effects of group, time, and the group × time interaction on short-term BP variability outcomes. An independent working correlation structure was specified, and robust standard errors were estimated using the sandwich estimator. No covariates were included in the primary models. Pairwise comparisons were assessed using the sequential Bonferroni test. This was a per-protocol analysis, including participants who completed at least 80% of the prescribed sessions and had valid baseline and endpoint ABPM recordings. Short-term BP variability requires each individual reading recorded throughout the 24-hour monitoring. Of the 63 participants who met the adherence criterion and completed both assessments, these individual readings were unavailable for 16, for whom only mean values were retained, because of problems in data export and a software failure that led to the loss of part of the recordings. Because angiotensin-converting enzyme (ACE) inhibitor use was imbalanced at baseline, a sensitivity analysis was performed by refitting all models with ACE inhibitor use entered both as a main effect and in interaction with time, retaining the group × time interaction. Statistical significance was defined as a two-sided *P* value <0.05. All analyses were conducted using SPSS Statistics version 18 (IBM Corp., Armonk, NY, USA). The sample size was determined for the primary outcomes of the parent trial. No *a priori* power calculation was performed for short-term BP variability.

## Results

The progress of participants through the phases of the study is presented in the CONSORT flow diagram (Figure 1), and Table 1 summarizes the reasons for discontinuation and for exclusion from the per-protocol analysis by study group. Among the 98 participants randomized in the RADAH trial, 12 in the CT-4 group and 9 in the CT-2 group discontinued the intervention because of severe floods in 2024, time constraints, family-related issues, or adverse events unrelated to the intervention. No participant discontinued because of an adverse event related to the intervention. A further 17 participants in the CT-4 group and 13 in the CT-2 group completed the intervention but were not included in the per-protocol analysis because their attendance was below 80% or because a valid ambulatory blood pressure recording was not available at both assessments. Accordingly, 20 participants in the CT-4 group and 27 in the CT-2 group were analyzed. Table 2 presents the clinical characteristics of the study participants. The use of angiotensin-converting enzyme inhibitors was more prevalent in the CT-4 group than in the CT-2 group (*P* = 0.015). No other significant differences were observed in the clinical or sociodemographic characteristics between the arms. Antihypertensive medication regimens remained unchanged throughout the 12-week intervention period in both groups. Attendance rates across the 12-week combined training intervention were similar between groups (*P* = 0.468), with values on average (SD) of 94.9 ± 5.1% in CT-2 and 93.6 ± 7.3% in CT-4. Out of the 24 planned sessions in CT-2, participants completed on average 23 ± 1 sessions, whereas in CT-4, out of 48 planned sessions, participants completed 45 ± 4 sessions. Participants included in the analysis did not differ from those not included in sociodemographic, anthropometric, ambulatory BP, or medication characteristics, except for peak VO_2_, which was lower among those not included (*P* = 0.022; Supplementary Table S2).

**Figure 1:**
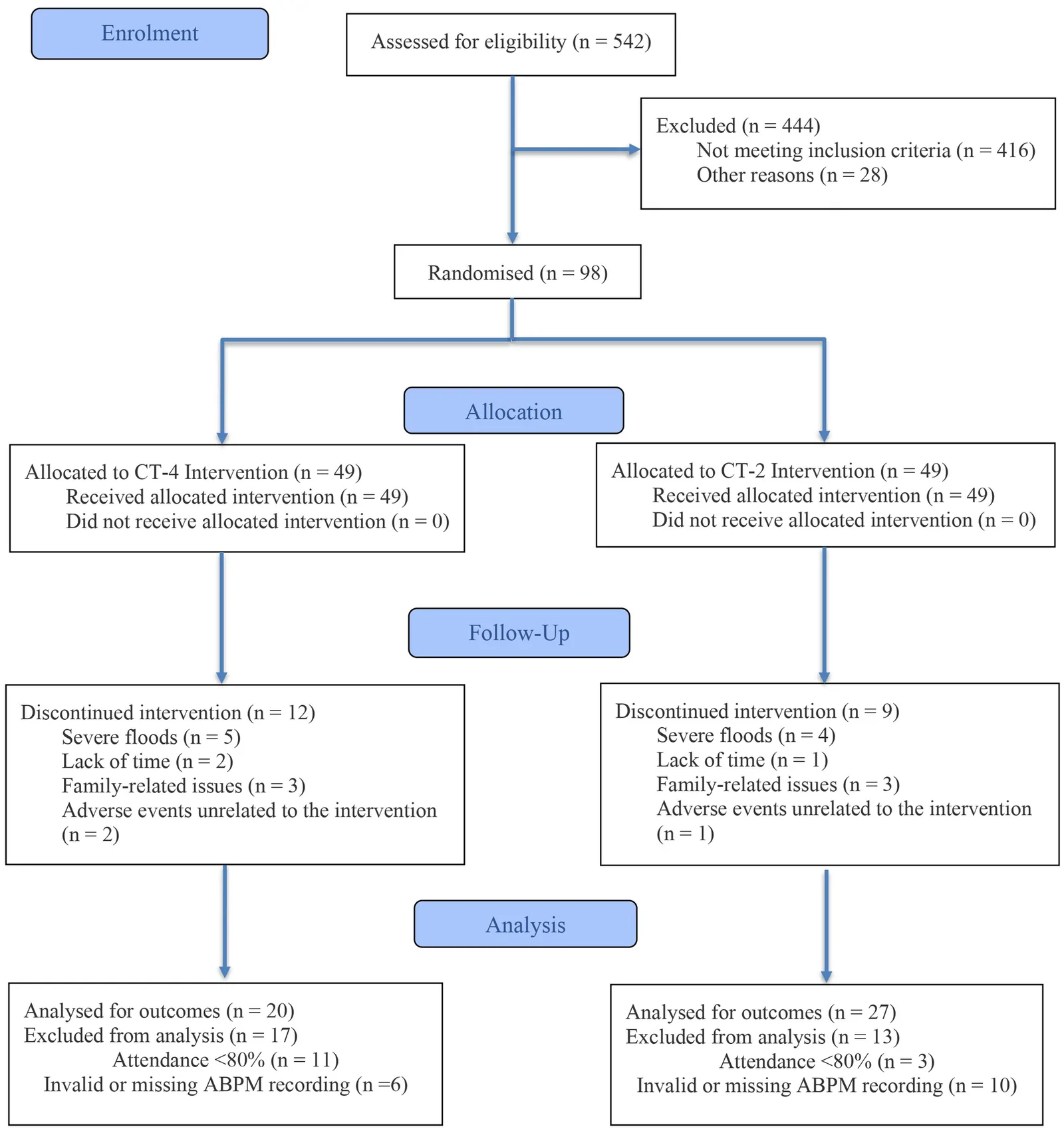
CONSORT flow diagram of the progress through the study phases.

**Table 1:** Reasons for discontinuation and exclusion from the per-protocol analysis, by study group.

Reason	CT-4 (*n* = 49)	CT-2 (*n* = 49)
**Discontinued the intervention**		
Severe floods (2024)	5 (10.2)	4 (8.2)
Lack of time	2 (4.1)	1 (2.0)
Family-related issues	3 (6.1)	3 (6.1)
Adverse events unrelated to the intervention	2 (4.1)	1 (2.0)
Femoral fracture	0 (0)	1 (2.0)
Pre-existing cervical spine condition	1 (2.0)	0 (0)
Ankle sprain at work	1 (2.0)	0 (0)
**Subtotal**	**12** (**24.5)**	**9** (**18.4)**
**Completed the intervention but excluded from the per-protocol analysis**		
Attendance < 80%	11 (22.4)	3 (6.1)
Invalid or missing ABPM recording	6 (12.2)	10 (20.4)
**Subtotal**	**17** (**34.7)**	**13** (**26.5)**
**Total not included in the per-protocol analysis**	**29** (**59.2)**	**22** (**44.9)**

Data are presented as n (%). Percentages were calculated based on the number of participants randomized to each study group (*n* = 49 per group). No participant discontinued because of an adverse event related to the intervention. CT-4, combined training performed four times per week; CT-2, combined training performed twice a week; ABPM, ambulatory blood pressure monitoring.

**Table 2:** Sociodemographic and clinical characteristics of the study participants.

Variables	CT-4 (*n* = 20)	CT-2 (*n* = 27)	*P* value
Ethnicity, *n* (%)			0.583
Black	4 (20)	4 (15)	
White	16 (80)	23 (85)	
Biological sex, *n* (%)			0.483
Female	10 (50)	17 (63)	
Male	10 (50)	10 (37)	
Age, years (mean ± SD)	65 ± 6	63 ± 8	0.456
Peak VO_2_, mL · kg^−1^ · min^−1^ (mean ± SD)	25.1 ± 4.8	28.6 ± 6.3	0.052
Body mass, kg (mean ± SD)	76.5 ± 12.2	77.3 ± 13.9	0.848
Height, m (mean ± SD)	1.62 ± 0.10	1.65 ± 0.07	0.366
Waist circumference, cm (mean ± SD)	98.1 ± 11.9	96.9 ± 10.8	0.732
BMI, kg/m^2^ (mean ± SD)	28.9 ± 3.4	28.4 ± 4.2	0.632
BMI classification, *n* (%)			0.151
Eutrophic	2 (10)	6 (22)	
Overweight	12 (60)	8 (30)	
Obesity	6 (30)	13 (48)	
Systolic BP (mmHg)			
Twenty-four hours	123 ± 10	122 ± 14	0.796
Daytime	127 ± 10	126 ± 13	0.947
Nighttime	114 ± 16	111 ± 17	0.526
Office	132 ± 10	131 ± 13	0.841
Diastolic BP (mmHg)			
Twenty-four hours	76 ± 6	75 ± 7	0.616
Daytime	79 ± 5	78 ± 7	0.816
Nighttime	70 ± 8	68 ± 8	0.356
Office	78 ± 9	75 ± 9	0.420
Antihypertensive medication, *n* (%)			
ACE inhibitors	10 (50)	5 (19)	0.015
ARBs	7 (35)	14 (52)	0.314
Diuretics	10 (50)	15 (56)	0.845
CCB	5 (25)	8 (30)	0.806
β blockers	2 (10)	9 (33)	0.074
Combined therapy	11 (55)	17 (63)	0.729
Diabetes medication, *n* (%)	4 (20)	4 (15)	0.583
Hypercholesterolemia medication, *n* (%)	10 (50)	17 (63)	0.483

Continuous variables are presented as mean ± standard deviation (SD), and categorical variables as absolute and relative (%) frequencies. CT-4, combined training performed four times per week; CT-2, combined training performed twice a week; BMI, body mass index; BP, blood pressure; ACE, angiotensin-converting enzyme inhibitors; ARBs, angiotensin II receptor blockers; CCB, calcium channel blockers.

Table 3 and Figure 2 present the ambulatory BP variability results assessed using the ARV and SD indexes. No significant group × time interaction was observed for systolic or diastolic ambulatory BP variability, regardless of the index adopted. There were no baseline differences between the CT-4 and CT-2 groups (*P* > 0.05 for all baseline comparisons). Significant reductions in 24-hour systolic ARV were observed in both the CT-4 (Δ: −2 ± 1; *P* = 0.003) and the CT-2 (Δ: −1 ± 1; *P* = 0.032) groups from baseline to endpoint assessments. A significant reduction in daytime systolic ARV was also observed in both the CT-4 (Δ: −3 ± 1; *P* = 0.008) and the CT-2 (Δ: −2 ± 1; *P* = 0.019) groups from baseline to endpoint assessments. An additional significant reduction in daytime diastolic ARV was observed in the CT-2 group (Δ: −1 ± 1; *P* = 0.016). No significant changes were observed for SD-based variability in any period. In the sensitivity analysis adjusted for ACE inhibitor use (Supplementary Table S1), the group × time interaction remained non-significant for eleven of the twelve variability outcomes, and the within-group reductions were unchanged. For 24-hour systolic ARV the interaction was significant (*P* = 0.046), a result that did not persist after accounting for multiple testing across outcomes (*P* ≥ 0.074). The interaction between time and ACE inhibitor use was not significant for any outcome (*P* ≥ 0.068).

**Table 3:** Short-term blood pressure variability at baseline and endpoint assessments.

Variables	CT-4 (*n* = 20)		CT-2 (*n* = 27)		Between-group difference (95% CI)	P value
	Baseline	Endpoint	Baseline	Endpoint		
**Twenty-four hours systolic BP variability**						
ARV (mmHg)	13 ± 1 (12–15)	11 ± 1 (10–12)^a^	12 ± 1 (10–12)	11 ± 1 (10–12)^a^	−1.1 (−2.8 to 0.5)	0.184
SD (mmHg)	17 ± 1 (16–20)	16 ± 1 (15–18)	16 ± 1 (15–17)	16 ± 1 (15–18)	−1.4 (−3.5 to 0.8)	0.209
**Daytime systolic BP variability**						
ARV (mmHg)	14 ± 1 (12–16)	11 ± 1 (10–13)^a^	13 ± 1 (11–14)	11 ± 1 (10–12)^a^	−1.1 (−3.2 to 0.9)	0.298
SD (mmHg)	16 ± 1 (14–19)	15 ± 1 (13–17)	15 ± 1 (14–16)	14 ± 1 (13–16)	−0.8 (−3.1 to 1.4)	0.473
**Nighttime systolic BP variability**						
ARV (mmHg)	12 ± 1 (11–13)	11 ± 1 (10–13)	11 ± 1 (10–12)	11 ± 1 (10–12)	−0.6 (−2.5 to 1.3)	0.530
SD (mmHg)	14 ± 1 (12–15)	14 ± 1 (12–15)	12 ± 1 (11–13)	12 ± 1 (11–14)	−0.2 (−2.3 to 1.8)	0.854
**Twenty-four hours diastolic BP variability**						
ARV (mmHg)	7 ± 1 (6–8)	7 ± 1 (6–8)	7 ± 1 (6–8)	8 ± 2 (5–11)	−1.4 (−4.7 to 1.8)	0.393
SD (mmHg)	10 ± 1 (9–12)	9 ± 1 (8–10)	10 ± 1 (9–11)	10 ± 1 (9–11)	0.1 (−1.6 to 1.6)	0.983
**Daytime diastolic BP variability**						
ARV (mmHg)	8 ± 1 (6–9)	7 ± 1 (6–8)	8 ± 1 (6–9)	7 ± 1 (5–7)^a^	0.5 (−1 to 2)	0.547
SD (mmHg)	9 ± 1 (8–10)	9 ± 1 (7–10)	9 ± 1 (8–10)	8 ± 1 (7–9)	0.8 (−1.2 to 2.7)	0.442
**Nighttime diastolic BP variability**						
ARV (mmHg)	6 ± 1 (5–7)	6 ± 1 (5–7)	6 ± 1 (5–7)	6 ± 1 (5–7)	−0.1 (−1.2 to 1)	0.867
SD (mmHg)	7 ± 1 (6–8)	8 ± 1 (7–9)	7 ± 1 (6–8)	7 ± 1 (6–8)	0.1 (−1.5 to 1.5)	0.998

Values are presented as mean ± standard error (95% confidence interval). Between-group difference refers to the difference in change from baseline to endpoint (CT-4 minus CT-2), with 95% confidence intervals. CT-4, combined training performed four times per week; CT-2, combined training performed twice a week; BP, blood pressure; ARV, average real variability; SD, standard deviation.

a Indicates statistical difference between baseline and endpoint assessments.

**Figure 2:**
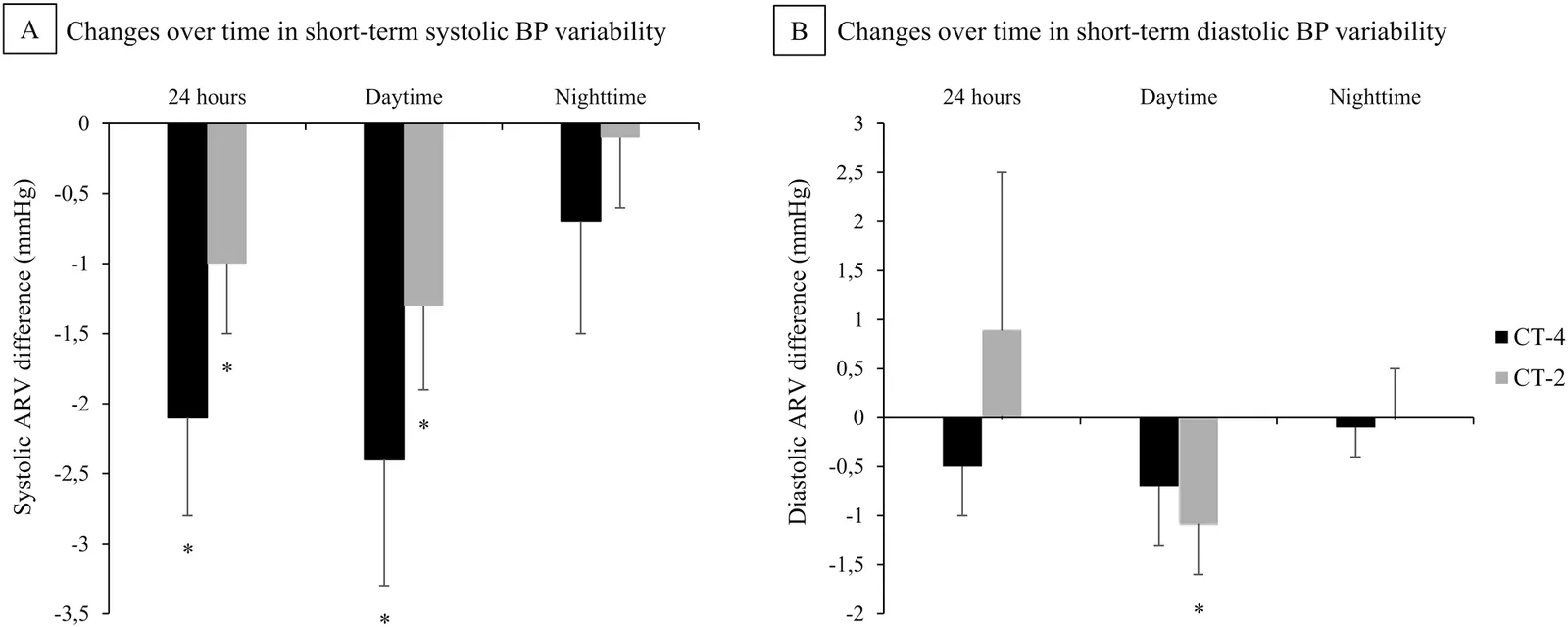
Changes over time in the average real variability of systolic **(A)** and diastolic **(B)** blood pressure after 12 weeks of combined training performed twice or four times per week. Data are presented as mean ± standard error; * indicates a significant difference between endpoint and baseline. BP, blood pressure; ARV, average real variability; CT-2, combined training performed twice a week; CT-4, combined training performed four times per week.

## Discussion

The main findings of this secondary analysis revealed that both twice-weekly and four-times-weekly combined training interventions reduced 24-hour and daytime systolic BP variability in older adults with hypertension. To date, no clinical trials have directly compared the effects of different weekly frequencies of exercise on short-term BP variability, and few studies have examined combined training in this context (8, 21). In the present study, after 12 weeks of combined training, reductions were observed in 24-hour systolic BP variability in both CT-4 (ARV: −2 mmHg) and CT-2 (ARV: −1 mmHg) arms, as well as in daytime systolic BP variability in CT-4 (ARV: −3 mmHg) and CT-2 (ARV: −2 mmHg), with no statistically significant difference detected between the frequencies (i.e., two vs. four). In comparison, a previous study reported that 12 weeks of combined training performed three times per week reduced 24-hour (ARV: −1.7 mmHg), daytime (ARV: −1.5 mmHg), and nighttime (ARV: −1.4 mmHg) systolic BP variability in older adults with hypertension (9). Despite differences in weekly training frequency, the total amount of exercise was similar between the present study (i.e., 120–160 min per week) and the other study (i.e., 160–180 min per week). In addition, the volume of aerobic and resistance exercise in the sessions of the combined training was similar between the studies, as well as the relative intensity prescribed. Another study reported that 10 weeks of combined training performed three times per week did not reduce short-term BP variability in postmenopausal women with hypertension or with normal BP levels (10). In this study, the amount of combined aerobic and resistance exercise per session was 20 min for each modality (i.e., 120 min per week), which was substantially lower than that reported in our study and in the aforementioned study (9). These findings suggest that total weekly exercise volume, rather than training frequency, may be a key determinant of the reduction in systolic BP variability. However, the present analysis was not powered to compare frequencies, and this interpretation remains hypothesis-generating.

The reductions in short-term BP variability were confined to the 24-hour and daytime periods, a pattern consistent with current evidence. A meta-analysis reported larger exercise effects on daytime than on night-time variability (11), and randomized trials found reduced daytime, but not night-time, systolic variability after aerobic (22) and combined training (23). The nocturnal BP pattern may further modulate this response, as combined training reduced 24-hour and daytime systolic variability in dippers but not in non-dippers, with between-group ARV changes (−1.0 to −1.3 mmHg) close to those observed here (23). In an observational cohort of older adults, each 2 mmHg higher 24-hour systolic ARV was associated with higher mortality and cardiovascular risk, independently of mean BP (24). However, whether exercise-induced reductions in short-term BP variability translate into improved cardiovascular outcomes remains uncertain. Therefore, although the reductions observed in the present study may indicate a potentially beneficial modification of a cardiovascular risk marker, our trial was not designed to evaluate clinical endpoints, and further intervention studies are needed to determine the clinical implications of changes in short-term BP variability.

Another factor to consider when interpreting the relationship between weekly training frequency and reductions in short-term BP variability is the association between post-exercise hypotension and chronic adaptations in BP control (25). Individuals who exercised more frequently may achieve better BP control than those who exercised less frequently, even when considering the same total amount of exercise per week (14). A previous study investigated the correlation between the acute and chronic effects of combined training on short-term BP variability after a single exercise session and following 10 weeks of combined training (26). Although a reduction in ARV was observed after the acute combined training session, this effect was not maintained after the chronic intervention. Thus, the absence of a correlation between the acute and chronic BP variability suggests that this outcome may be more strongly related to the total amount of exercise performed than to weekly training frequency alone, reinforcing the findings of the present study.

Reductions in daytime diastolic BP variability were observed after the combined training intervention performed twice a week (daytime ARV: −1 mmHg). Some studies reported a reduction in short-term systolic BP variability but not in diastolic BP (9, 27). Another reported a reduction in both systolic and diastolic BP variability (28), and another reported a reduction only in diastolic BP variability (29). The heterogeneity of exercise protocols across studies, combined with the lack of trials specifically investigating the mechanisms underlying changes in systolic and diastolic BP variability, limits direct comparison and prevents a clear understanding of the diastolic BP variability findings across the studies. We hypothesize that longer exercise sessions, such as those performed in the CT-2 group, may have promoted greater adaptations in peripheral vascular resistance, thereby contributing to improvements in BP regulation (29). Pooled data indicate that combined training can reduce diastolic BP variability (11), so this finding is not isolated. However, it was confined to one arm without a group × time interaction and may reflect the multiplicity of within-arm comparisons rather than a frequency-specific effect. Mechanistically, the daytime systolic reduction is compatible with improved arterial buffering and central hemodynamics, since aerobic training lowers central systolic pressure alongside daytime variability (22). The autonomic contribution is less certain, as aerobic training can improve autonomic modulation without measurable changes in BP variability (30).

Although the CT-4 group had a higher prevalence of ACE inhibitor use and a borderline higher peak VO_2_ at baseline, office BP, ambulatory BP, and short-term BP variability were comparable between groups before the intervention and at baseline measurements, respectively. Moreover, antihypertensive medication regimens remained unchanged throughout the study, and reductions in short-term BP variability were observed in both groups. The sensitivity analysis did not provide evidence that ACE inhibitor use meaningfully affected the observed changes in short-term BP variability. Nevertheless, the per-protocol selection of participants after randomization may have introduced selection bias, and residual confounding cannot be excluded (31). These reductions should also be considered against the measurement properties of ARV. In hypertensive patients undergoing two ambulatory recordings 4 weeks apart, 24-hour systolic ARV showed moderate reproducibility (ICC 0.618) and daytime systolic ARV poor reproducibility (ICC 0.460), with mean differences between recordings of 0.1 and 0.4 mmHg (32). The reductions observed here exceed these group-level differences, but it remains uncertain whether a change of 1–3 mmHg exceeds measurement variability in an individual patient.

This study has some limitations that should be acknowledged to properly interpret its results. This is a secondary analysis of a previously published randomized clinical trial that included only participants with an attendance rate of at least 80%. Because participants were selected based on adherence after randomization, this analysis is per-protocol in nature and was not powered to detect between-frequency differences in BP variability. Consequently, the non-significant group × time interaction should be interpreted as an absence of evidence rather than evidence of equivalence. Furthermore, restricting the analysis to participants who adhered to the intervention may have partially compromised the benefits of randomization and introduced the possibility of selection bias. Although this selective inclusion may have reduced the sample size and limited the statistical power, it allowed us to evaluate the effect of different weekly training frequencies among participants who adhered to the intervention protocol. Participants not included in the analysis had lower baseline cardiorespiratory fitness, which may limit the generalizability of the findings to less fit individuals. For part of the adherent participants, only mean ABPM values were available, without the individual readings recorded throughout the 24-hour monitoring, which reduced the sample size and precluded an intention-to-treat analysis of short-term BP variability. Finally, although a sensitivity analysis accounting for ACE inhibitor use did not change the findings, the baseline imbalance in antihypertensive medication and the modest sample size mean that residual confounding cannot be excluded. Therefore, these findings should be interpreted in light of the inherent limitations of a per-protocol analysis.

Furthermore, the absence of a non-exercising control group limits the ability to determine the extent to which the observed reductions in short-term BP variability were attributable to the combined training intervention itself, as opposed to other factors such as regression to the mean or temporal variation. Additionally, the relatively short intervention period (12 weeks) limits the assessment of longer-term exercise-induced adaptations and precludes direct comparisons with other exercise modalities. The strengths of this study include its comprehensive assessment of short-term BP variability using ARV and SD indices, which provides a framework for future research and evidence synthesis. Additionally, the pragmatic combined training intervention is easily scalable to real-world environments due to its minimal infrastructure requirements. Lastly, this work provides critical insights into the clinical impact of exercise frequency, helping to optimize non-pharmacological strategies for hypertension management.

## Conclusion

In conclusion, 12 weeks of combined training performed twice or four times per week with the same total weekly volume was not associated with statistically significant differences in short-term BP variability between training frequencies among aging adults with hypertension. Although reductions in short-term systolic BP variability were observed in both training groups, this study did not detect evidence of a difference between training frequencies.

## Data Availability

The original contributions presented in the study are included in the article/Supplementary Material, further inquiries can be directed to the corresponding author.
