# Supplementary material for: Effects of different weekly frequencies of combined training on blood pressure variability in aging adults with hypertension
Source: Front Rehabil Sci. 2026 Oct 19;7:1925760. doi: 10.3389/fresc.2026.1925760 (PMC13724428; doi:10.3389/fresc.2026.1925760)
Supplement: Supplementary file 1 [file Table1.docx]

**Table S1. Sensitivity analysis adjusted for angiotensin-converting enzyme (ACE) inhibitor use**

| **Outcome** | **Group** | **Time** | **ACE** | **Time × ACE** | **Group × Time** | **Group × Time (unadjusted)** |
| --- | --- | --- | --- | --- | --- | --- |
| **24-hour systolic BP variability** |  |  |  |  |  |  |
| ARV | .261 | .002 | .935 | .068 | .046 | .184 |
| SD | .285 | .295 | .179 | .630 | .140 | .209 |
| **Daytime systolic BP variability** |  |  |  |  |  |  |
| ARV | .468 | .006 | .782 | .123 | .072 | .298 |
| SD | .253 | .139 | .433 | .204 | .156 | .473 |
| **Nighttime systolic BP variability** |  |  |  |  |  |  |
| ARV | .087 | .599 | .505 | .330 | .376 | .530 |
| SD | .044 | .725 | .086 | .742 | .791 | .854 |
| **24-hour diastolic BP variability** |  |  |  |  |  |  |
| ARV | .255 | .507 | .161 | .171 | .255 | .393 |
| SD | .492 | .333 | .950 | .589 | .862 | .983 |
| **Daytime diastolic BP variability** |  |  |  |  |  |  |
| ARV | .990 | .099 | .437 | .169 | .896 | .547 |
| SD | .852 | .274 | .625 | .463 | .585 | .442 |
| **Nighttime diastolic BP variability** |  |  |  |  |  |  |
| ARV | .543 | .948 | .820 | .432 | .656 | .867 |
| SD | .759 | .485 | .784 | .377 | .746 | .998 |

ARV: average real variability; SD: standard deviation; ACE: angiotensin-converting enzyme. Group: combined training performed twice versus four times per week.
